# Formation and suppression of hydrogen blisters in tunnelling oxide passivating contact for crystalline silicon solar cells

**DOI:** 10.1038/s41598-020-66801-4

**Published:** 2020-06-15

**Authors:** Sungjin Choi, Ohmin Kwon, Kwan Hong Min, Myeong Sang Jeong, Kyung Taek Jeong, Min Gu Kang, Sungeun Park, Kuen Kee Hong, Hee-eun Song, Ka-Hyun Kim

**Affiliations:** 1 Photovoltaics Laboratory, Korea Institute of Energy Research, Daejeon, 34129 South Korea; 2 Department of Physics, Chungbuk National University, Cheongju, 28644 Chungbuk South Korea; 3 Department of Materials Science and Engineering, Korea University, Seoul, 02841 South Korea; 4 Solar Cell R&D Team, R&D Center, Shinsung E&G Co., Ltd., Seongnam, 13543 South Korea; 5 Research Institute for Nanoscale Science and Technology, Cheongju, 28644 Chungbuk South Korea

**Keywords:** Solar cells, Surfaces, interfaces and thin films

## Abstract

The formation of hydrogen blisters in the fabrication of tunnelling oxide passivating contact (TOPCon) solar cells critically degrades passivation. In this study, we investigated the formation mechanism of blisters during the fabrication of TOPCons for crystalline silicon solar cells and the suppression of such blisters. We tested the effects of annealing temperature and duration, surface roughness, and deposition temperature on the blister formation, which was suppressed in two ways. First, TOPCon fabrication on a rough surface enhanced adhesion force, resulting in reduced blister formation after thermal annealing. Second, deposition or annealing at higher temperatures resulted in the reduction of hydrogen in the film. A sample fabricated through low-pressure chemical vapor deposition at 580 °C was free from silicon–hydrogen bonds and blisters after the TOPCon structure was annealed. Remarkably, samples after plasma-enhanced chemical vapor deposition at 300, 370, and 450 °C were already blistered in the as-deposited state, despite low hydrogen contents. Analysis of the hydrogen incorporation, microstructure, and deposition mechanism indicate that in plasma-enhanced chemical vapor deposition (PECVD) deposition, although the increase of substrate temperature reduces the hydrogen content, it risks the increase of porosity and molecular-hydrogen trapping, resulting in even more severe blistering.

## Introduction

The conversion efficiency of industrial crystalline silicon (c-Si) solar cells has continuously improved and is about to reach 22%^1^. The current method of wafer-surface passivation to improve efficiency is to reduce recombination losses at metal contacts by using small-area local contacts on the rear surface while the rest of the surface is passivated. The passivated emitter and rear contact (PERC) structure is a substantial upgrade over the conventional back surface field (BSF) structure and is employed in the industry as a new standard^2^. However, the recombination loss at the metal contacts is expected to be a major problem once the conversion efficiency reaches 23%^3^. Therefore, passivating contacts are spotlighted as an important research topic to realise a technical improvement in solar-cell technology. A passivating contact has been regarded as an ideal solar-cell structure since the 1980s^4,5^. After early industrial implementation by SunPower Corporation in 2010^6^, the passivating-contact structure was extensively studied. The idea of a passivating contact is the formation of a full-area contact without direct contact of the wafer and absorber material with the metal electrode. The most intensively studied passivating-contact structure is heavily doped polycrystalline silicon (poly-Si) on silicon oxide (tunnelling oxide or SiO_x_), which is called a tunnelling oxide passivating contact (TOPCon)^7^ or polycrystalline silicon on oxide (POLO)^8,9^.

In this passivating-contact structure, heavily doped poly-Si can be either deposited through low-pressure chemical vapor deposition (LPCVD)^9–11^ or crystallised by the thermal annealing of hydrogenated amorphous silicon (a-Si:H) deposited through plasma-enhanced chemical vapor deposition (PECVD)^12–15^. In either case, the layer stack structure must be thermally annealed at a temperature of approximately 850 °C to reduce interfacial defects^13,16^.

One of the problems in TOPCon structures fabricated through the PECVD of an a-Si:H layer on top of a tunnelling-oxide layer is blister formation, particularly during thermal annealing^13,14^. Blisters are formed because of hydrogen-rich precursor gases and the deposition conditions. The “hydrogenated” a-Si:H thin film releases its hydrogen content of 10^20^~10^22^ cm^−3^ during high-temperature annealing. A portion of released hydrogen accumulates at the interface between a-Si:H and SiO_x_, eventually forming blisters. Blister formation degrades the TOPCon quality^13,14^ owing to the inhomogeneity of film morphology and increase of the lateral transport path. Therefore, the fabrication process must be fine-tuned to solve the issue of blistering.

Despite the significance mentioned above, the formation mechanism and suppression of blisters during the fabrication of TOPCon are not yet fully understood. In this study, we investigated these aspects. We tested the effects of various factors in fabrication, such as the annealing temperature and duration, surface roughness, and deposition temperature. Blister formation in TOPCon was suppressed by introducing surface roughness and reducing the hydrogen content of the film by depositing or annealing at higher temperatures.

## Results and discussion

### Effect of annealing conditions

**Figure 1:**
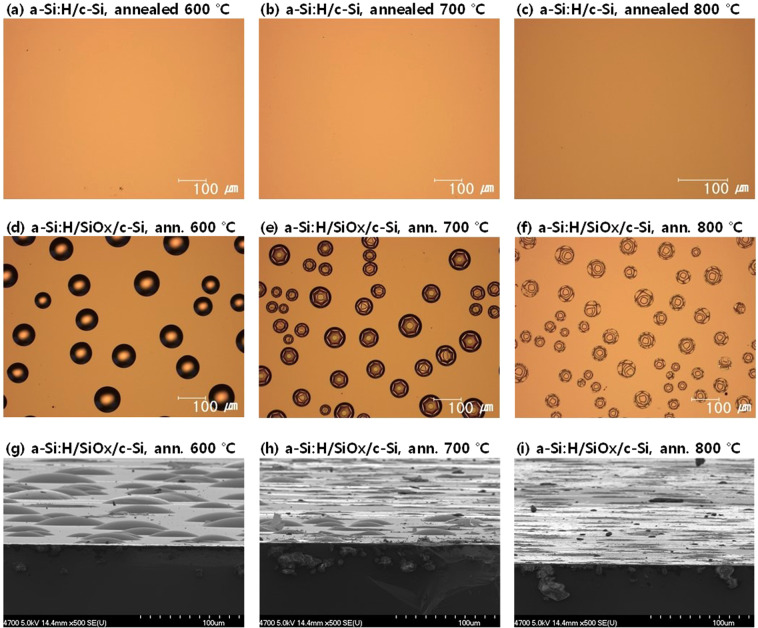
Optical images of the surface of (**a–c**) a-Si:H/c-Si and (**d–f**) a-Si:H/SiO_x_/c-Si structures. SEM images of (**g–i**) a-Si:H/SiO_x_/c-Si structures annealed at 600, 700, and 800 °C. CMP-treated semiconductor-grade c-Si was used as the substrate.

Fig. 1 shows a series of optical images of the surface of a-Si:H films deposited without an oxide layer (a-Si:H/c-Si, Fig. 1a–c) and a-Si:H films deposited on tunnelling-oxide layers (a-Si:H/SiO_x_/c-Si, Fig. 1d–f), as well as scanning electron microscopy (SEM) images of a-Si:H/SiO_x_/c-Si structures (Fig. 1g–i). For this series, the samples are deposited through PECVD, and chemical–mechanical polishing (CMP)-treated semiconductor-grade c-Si was used as the substrate.

The optical images of the sample surface were taken after annealing at 600, 700, and 800 °C. The a-Si:H/c-Si structures showed no blistering after annealing, irrespective of the annealing temperature. On the other hand, the a-Si:H/SiO_x_/c-Si structures showed significant blistering after annealing. Blisters appear as circular objects on the a-Si:H surface in optical microscopic images. Furthermore, the shape of blisters was cross verified by tilting the SEM view. It is worth noting that the fracture of blisters is observed at annealing temperatures of 700 and 800 °C, while the blisters at 600 °C appear to retain their shape. Because the a-Si:H films fabricated via PECVD are annealed in a belt furnace for a short duration of 5 min, annealing at a high temperature of 700 or 800 °C would also introduce a very high heating rate. Hydrogen effusion under such rapid heating would build a volcanic gas pressure of molecular hydrogen in the blisters, possibly resulting in the fracture of blisters at high annealing temperatures.

**Figure 2:**
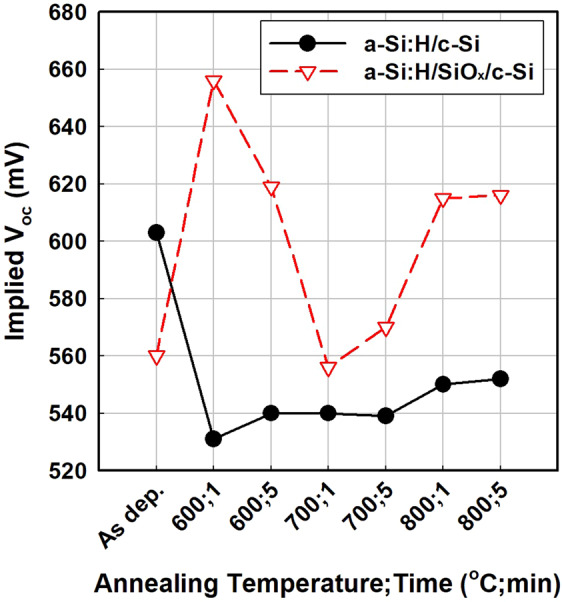
Implied V_oc_ of a-Si:H/c-Si and a-Si:H/SiO_x_/c-Si structures annealed at various temperatures for different durations. CMP-treated semiconductor-grade c-Si was used as the substrate.

As shown in Fig. 1, the a-Si:H/c-Si sample, without an oxide layer, showed no blister formation. The reason for this result pertains to the hydrogen transport mechanism of SiO_x_/c-Si, which has been studied for metal-oxide semiconductors (MOSs)^17^. Nickel showed that the oxide layer in the MOS structure reduces the hydrogen flux into the underlying layers (c-Si), thereby functioning as hydrogen diffusion barrier^17^. Therefore, in the case of a-Si:H/SiO_x_/c-Si, hydrogen accumulates at the a-Si:H/SiO_x_ interface and forms blisters, while hydrogen can penetrate into the c-Si substrate in the case of a-Si:H/c-Si. In other words, the primary cause of blistering is SiO_x_, and the blistering can be avoided by removing SiO_x_. However, the removal of SiO_x_ is not a feasible solution for blister prevention, because the tunnelling-oxide layer is necessary to realise a passivating contact. Figure 2 shows the evolution of implied V_oc_ of a-Si:H/c-Si and a-Si:H/SiO_x_/c-Si after annealing at various temperatures and durations, shown in Fig. 1. The a-Si:H/c-Si sample shows low implied V_oc_ overall, except in the as-deposited state. In the as-deposited state, n-type a-Si:H fabricated via PECVD works as a good passivation layer, providing both chemical and field-effect passivation^16^. Dehydrogenation and crystallisation during annealing introduce excess defects at the interface, resulting in the poor implied V_oc_ of the a-Si:H/c-Si sample after annealing.

For a-Si:H/SiO_x_/c-Si, the sample with the tunnelling-oxide layer, it is notable that the implied V_oc_ is high for annealing at 600 °C for 1 min as well as at 800 °C for 1 and 5 min. Such behaviour can also be found in the literature^15,16^ and is attributed to the change of passivation mechanism. Annealing at 600 °C for 1 min causes the rearrangement of silicon–hydrogen bonds at interfaces; consequently, chemical passivation by the silicon–hydrogen bond is dominant^15^. At higher temperatures, silicon–hydrogen bonds rupture, and the hydrogen in the sample effuses; meanwhile, a-Si:H crystallises to poly-Si, and the tunnelling oxide is reconstructed. Therefore, at temperatures above 800 °C, a carrier-selective contact is formed, allowing only electrons to be transferred while holes are expelled so that electron–hole recombination is fundamentally avoided^15,16^. However, the overall implied V_oc_ of the a-Si:H/SiO_x_/c-Si shown in Fig. 2 appears to be poor compared to results reported elsewhere^7,9,12,13,15,16^. It is believed that the deposition and annealing processes of the thin film should be further optimised and blister formation should be suppressed to improve the passivation quality.

**Figure 3:**
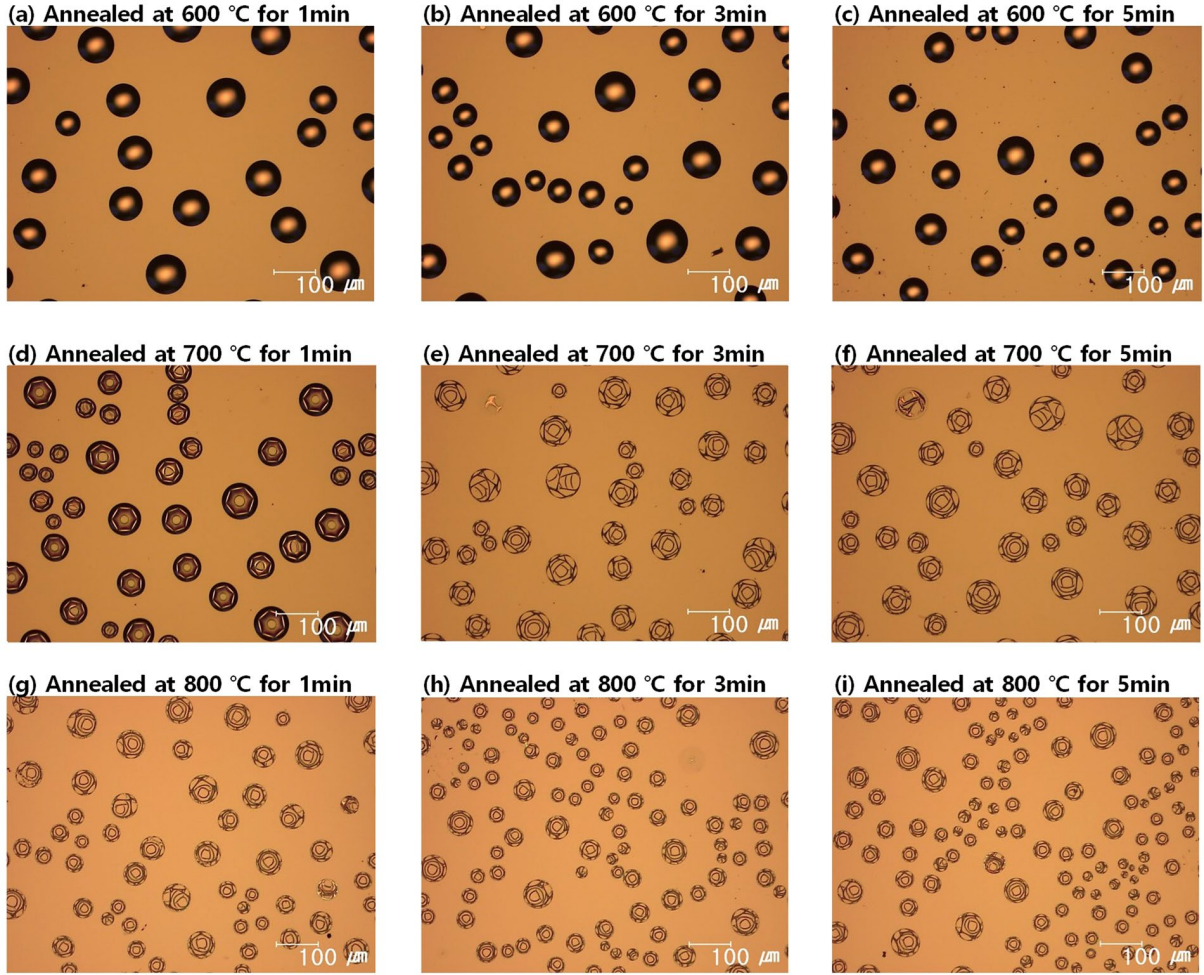
Optical images of the surface of a-Si:H/SiO_x_/c-Si layer stacks after annealing at various temperatures for different durations: (**a**) 600 °C for 1 min, (**b**) 600 °C for 3 min, (**c**) 600 °C for 5 min, (**d**) 700 °C for 1 min, (**e**) 700 °C for 3 min, (**f**) 700 °C for 5 min, (**g**) 800 °C for 1 min, (**h**) 800 °C for 3 min, and (**i**) 800 °C for 5 min.

**Figure 4:**
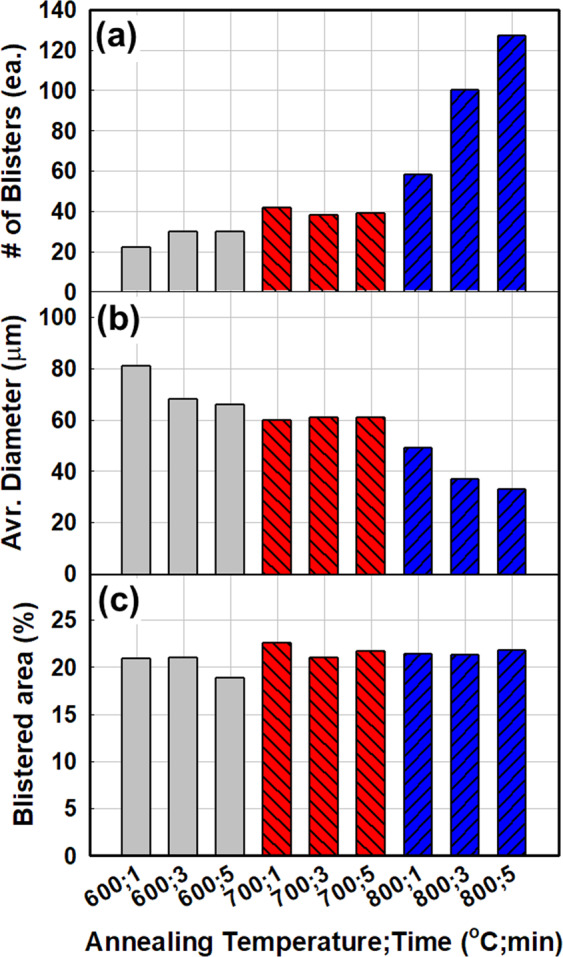
Statistics of blister formation based on the optical images presented in Fig. 3: (**a**) The number of blisters, (**b**) average diameter of blisters, and (**c**) total blistered area for different annealing temperatures and durations are shown.

In order to test the effects of annealing conditions on the blister formation, the a-Si:H/SiO_x_/c-Si layer stack was annealed at various temperatures and for different durations. Figure 3 shows optical images of the surface of the a-Si:H/SiO_x_/c-Si layer stacks annealed at various temperatures and for different durations, and Fig. 4 shows the statistics of blister formation obtained by analysing the optical images shown in Fig. 3 with ImageJ software. According to the general trend of the results, a higher number of blisters are formed at higher annealing temperatures and longer annealing durations, while larger blisters are formed at lower annealing temperatures and shorter annealing durations. Figure 4(c) shows the total blistered areas in the optical images. Regardless of the annealing condition, i.e., the number of blisters and blister size, the blistered area is always approximately 20% of the entire surface area. Blistering should be related to the effusion rate of hydrogen from silicon–hydrogen bonds because blisters are formed by the detachment of interface by accumulated hydrogen. In other words, blistering relies on the hydrogen effusion from the a-Si:H and cannot be avoided by controlling the annealing condition. However, if the temperature ramp rate is extremely low, blistering could be discouraged because of the slow resulting effusion rate of hydrogen. de Calheiros Velozo *et al*. tested the thermal dehydrogenation of an a-Si:H film deposited on a c-Si wafer and showed that blistering is absent if the heating rate is as low as 0.5 °C/min^18^. Therefore, the blistering could be prevented under an extremely low heating rate, but such a low heating rate would be impractical and difficult to implement in the industry, considering the throughput of modern solar-cell manufacturing processes. Therefore, the blister formation should be suppressed by either modifying the adhesion between a-Si:H and SiO_x_ or by controlling the hydrogen content of a-Si:H.

### Effect of surface roughness

**Figure 5:**
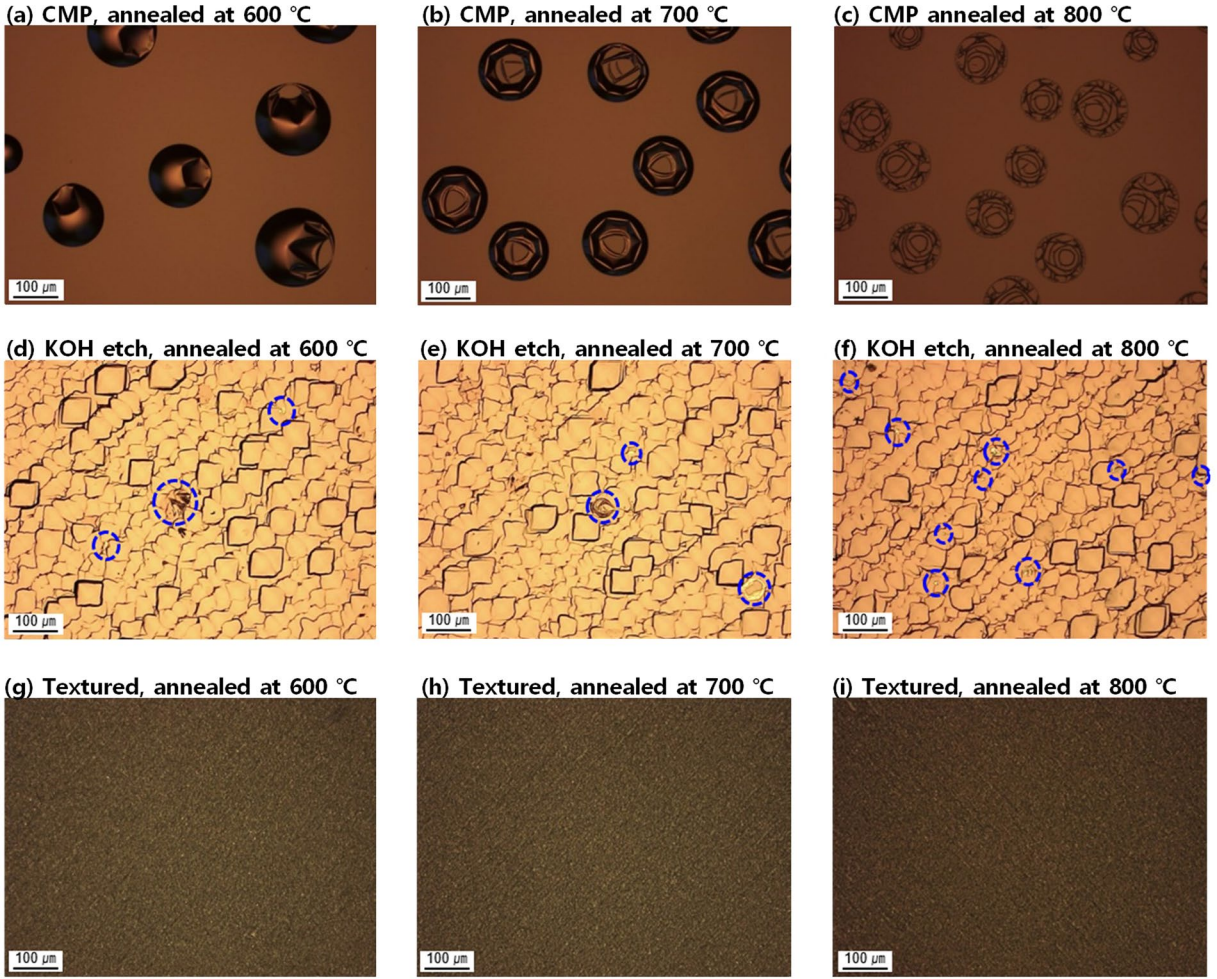
Optical images of the surface of TOPCons fabricated on various silicon wafer surfaces: (**a–c**) chemically mechanically polished surfaces; (**d–f**) surfaces etched in KOH; and (**g–i**) pyramid-textured surfaces. The blisters on KOH-etched samples are marked with blue circles.

Fig. 5 shows a series of optical images of the surface of TOPCon after thermal annealing. The contacts were fabricated on various silicon wafers: a wafer treated with CMP, a wafer etched in potassium hydroxide (KOH), and a pyramid-textured wafer. The wafer treated with CMP shows the most significant blister formation after thermal annealing. The KOH-etched c-Si surface shows surface roughness, which originated from the crystallographic character of c-Si. The TOPCon fabricated on the KOH-etched surface shows a significantly reduced number of blisters, as marked with blue circles. Interestingly, the TOPCon fabricated on the textured surface shows no visible blister formation after thermal annealing. This result suggests that the introduction of surface roughness suppresses blister formation. Peng and Chen theoretically investigated the peeling behaviour of a thin film adhered to a corrugated substrate^19^. They found that the maximal peel-off force (adhesion) of the corrugated interface increases as the substrate roughness increases. Because the corrugated structure can be regarded as a simplified two-dimensional model of surface roughness, the adhesion force of the thin film would increase as the surface roughness increases. Therefore, the fabrication of TOPCon on a rough surface would prevent blister formation. Indeed, recent research in the industry showed that industrial TOPCon solar cells can be fabricated using a textured back surface^3^. TOPCon fabrication on a textured back surface would also be beneficial for bifacial solar-cell applications^3^.

**Figure 6:**
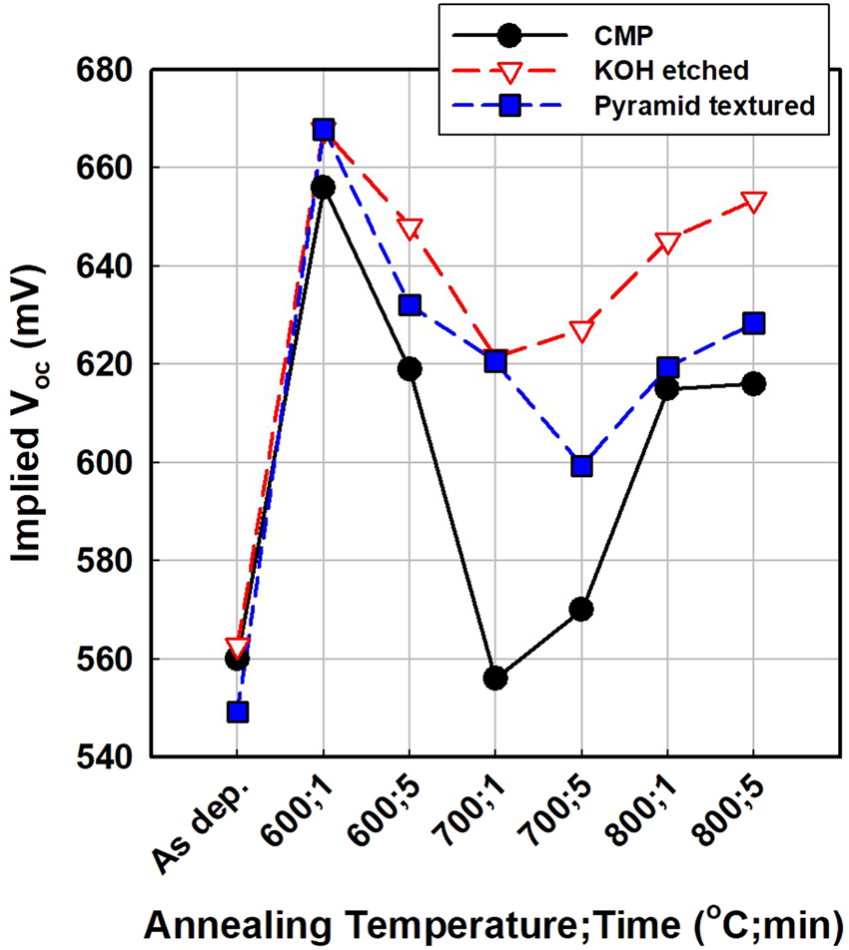
Implied V_oc_ of a-Si:H/SiO_x_/c-Si structures fabricated on CMP, KOH-etched, and pyramid-textured c-Si surface annealed at various temperatures for different durations.

Figure 6 shows the evolution of implied V_oc_ of the TOPCons fabricated on the CMP-treated, KOH-etched, and pyramid-textured c-Si surfaces. In agreement with the optical images shown in Fig. 5, the implied V_oc_ of the contact fabricated on the CMP-treated surface, which showed the most significant blistering, was the lowest throughout the range of thermal annealing conditions. It is worth noting that the pyramid-textured sample shows a lower implied V_oc_ than the KOH-etched sample. It is believed that further optimisation is needed to fabricate TOPCons on pyramid-textured surfaces. Improved passivation is expected on the pyramid-textured sample after optimisation considering factors such as the increased surface area after texturing, variation in the effective thin-film thickness with deposition on inclined planes, different crystallographic orientations on the <111> pyramid plane and the <100> flat plane, and conformal coverage issue of both SiO_x_ and a-Si:H on the pyramid structure.

### Effect of substrate temperature and hydrogen content of deposition

Controlling the substrate temperature during deposition modifies the hydrogen content of the silicon thin film^20,21^. In the case of PECVD, the growing film consists of silicon–hydrogen bonds at substrate temperatures below 400 °C^22^, and the hydrogen content of the resulting film is in the range of 10–15 at.%^21^. At higher substrate temperatures, hydrogen acquires sufficient kinetic energy to become volatile^23^. In other words, a-Si:H with a low hydrogen content would be obtained at a high substrate temperature. Monosilane (SiH_4_) starts to thermally decompose above 420 °C, and the decomposition becomes significant at approximately 550 °C^24^. Therefore, a completely dehydrogenated thin film should be obtained if the substrate temperature is higher than 550 °C.

**Figure 7:**
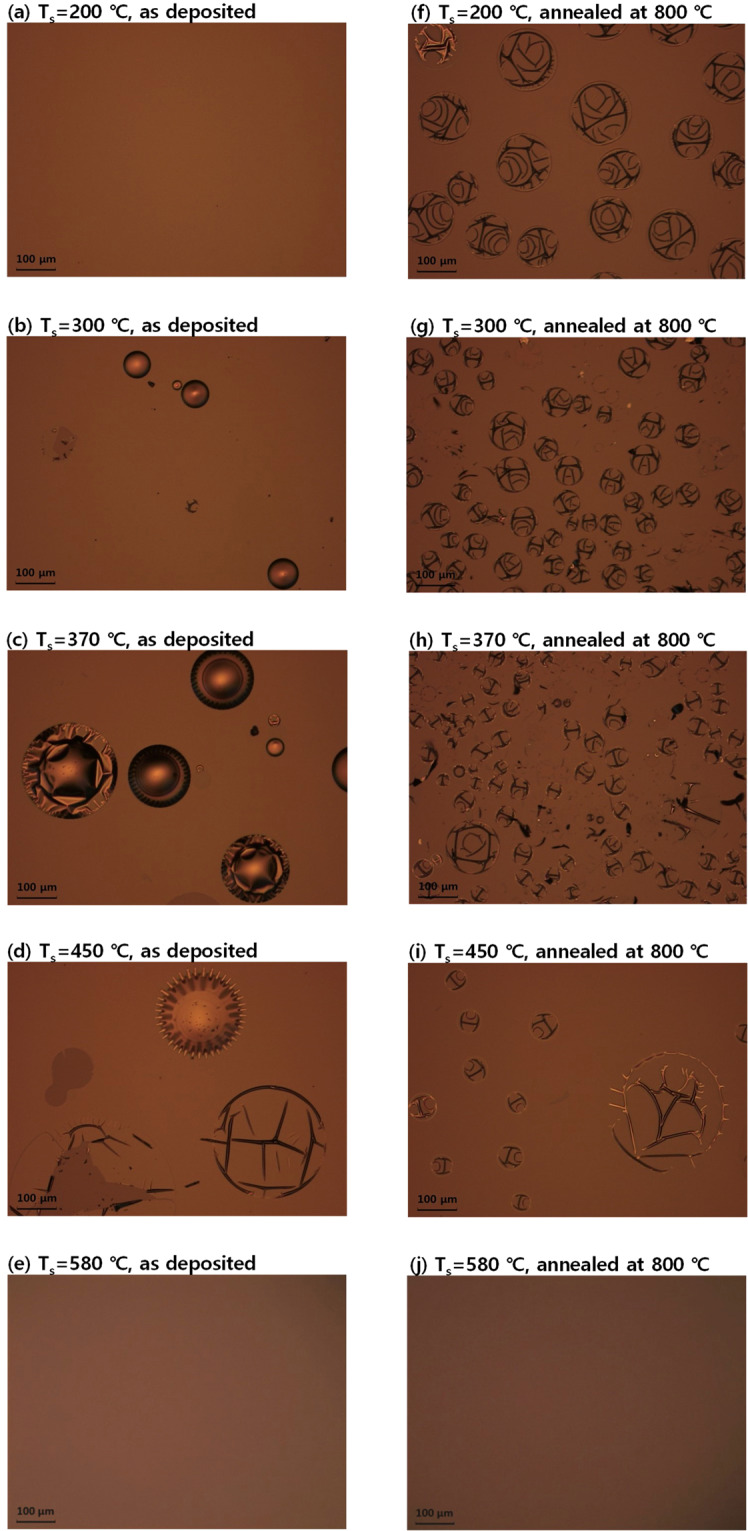
Optical images of the surface of TOPCons (**a–e**) before and (**f–j**) after annealing. The samples were deposited at (**a**,**f**) 200 °C; (**b,g**) 300 °C; (**c**), (h) 370 °C; (**d,i**) 450 °C; and (**e**,**j**) 580 °C.

Figure 7 shows a series of optical images of the a-Si:H/SiO_x_/c-Si layer stack, fabricated at various substrate temperatures, before and after thermal annealing. In order to test the blistering behaviour of the sample deposited at a high temperature to ensure complete dehydrogenation, we added a sample fabricated via LPCVD in the temperature series. Spectroscopic ellipsometry measurement and modelling revealed that the LPCVD film has a low crystalline volume fraction; thus, the LPCVD deposited sample was polycrystalline silicon (poly-Si). The poly-Si fabricated via LPCVD at a substrate temperature of 580 °C appears to be free from blistering after thermal annealing. This result suggests that the deposition of a silicon thin film at temperatures above 580 °C can prevent blistering in TOPCon fabrication.

Deposition at high temperature may yield a poor-quality material because the growing surface is no longer covered by silicon–hydrogen bonds^21,25^. The loss of surface hydrogen coverage introduces dangling bonds on the surface, resulting in the short surface diffusion of the precursor and, in turn, elevated porosity and roughness of the grown film^25^. The temperature series shows that samples fabricated via PECVD at 300, 370, and 450 °C are already blistered in the as-deposited state. The reason for this result can be twofold. First, as explained above, a high substrate temperature reduces the surface hydrogen coverage of the growing surface, introducing depressed surface diffusion of precursor gas^21,25^. Therefore, the increase of substrate temperature would have resulted in an increased concentration of voids, which can trap molecular hydrogen. The second reason pertains to the effusion of hydrogen. As the substrate temperature ramps up, the elimination probability of surface hydrogen increases. The film deposition process involves the elimination of a hydrogen atom from one silicon atom by the molecular silane species. At a high temperature, direct hydrogen evolution (effusion) from the bulk of the film becomes significant. The hydrogen evolution is, therefore, molecular hydrogen elimination, in which one hydrogen atom moves over a dimer to the second hydrogen atom and then forms a hydrogen molecule^21^. The process is similar to the case of molecular hydrogen evolution at the hydrogenated surface of <100> c-Si. The hydrogen effusion is indeed an isotropic process; therefore, a portion of hydrogen may be trapped at the interface between the growing film and substrate, resulting in blisters in the as-deposited state. It should also be pointed out that the fine-tuning of PECVD parameters, such as the relative gas flow ratio, can suppress blistering^26^. However, in this study, we tested the effect of substrate temperature at a constant gas flow ratio.

**Figure 8:**
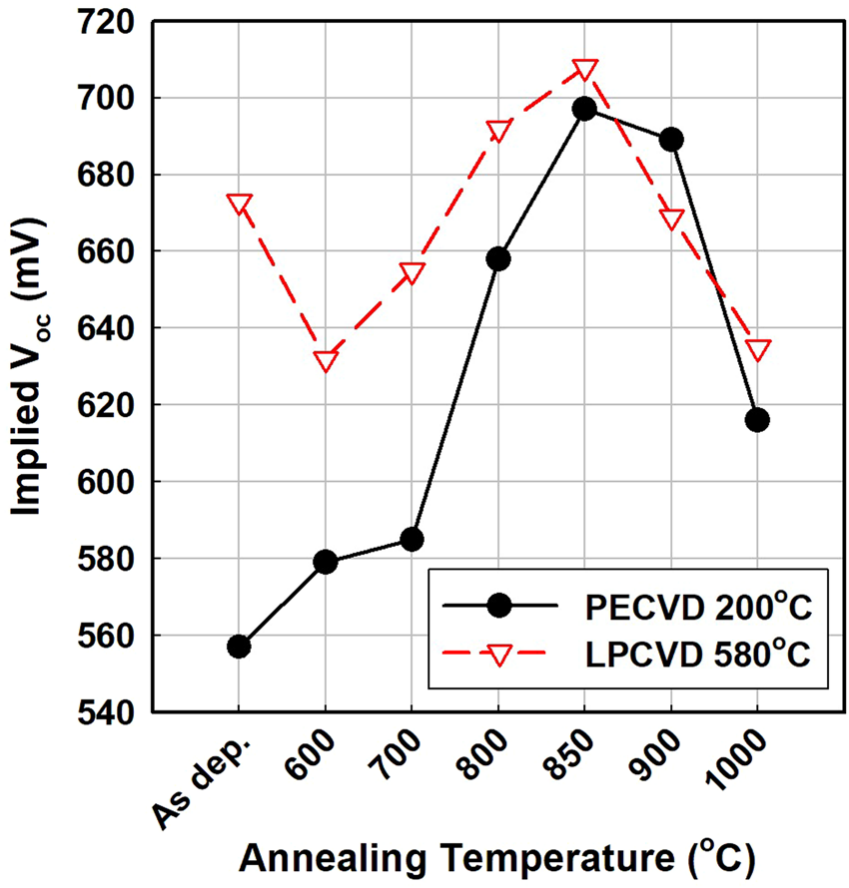
Evolution of implied V_oc_ of TOPCons fabricated via PECVD at 200 °C and LPCVD at 580 °C upon annealing at various temperatures. Both PECVD a-Si:H and LPCVD poly-Si are fabricated on KOH-etched solar-grade wafers; thus, blister formation was significantly reduced.

Figure 8 shows the implied V_oc_ of two TOPCons, the a-Si:H fabricated via PECVD at 200 °C and the poly-Si fabricated via LPCVD at 580 °C, in the as-deposited state and after annealing at various temperatures. Both PECVD a-Si:H and LPCVD poly-Si are fabricated on KOH-etched solar-cell–grade wafers; thus, blister formation was already significantly reduced. The TOPCon using LPCVD poly-Si at 580 °C shows a higher implied V_oc_ than that using PECVD a-Si:H at 200 °C for all annealing temperatures except 900 °C. Nevertheless, the PECVD a-Si:H sample showed a significantly improved implied V_oc_ compared to the result in Fig. 2, suggesting that use of a rough surface enhanced passivation. The decrease in the implied V_oc_ after annealing at temperatures above 900 °C can be attributed to the change in stoichiometry of the tunnelling oxide to form silicon suboxide, degrading passivation.

As mentioned above, deposition at different substrate temperatures changes the hydrogen content of the thin film. The experimental results presented above indicate that blistering can be avoided if we deposit the silicon thin film at a high temperature such as 580 °C. Furthermore, the TOPCon deposited at 580 °C showed superior passivation quality to that deposited at 200 °C. In order to test behaviour related to silicon–hydrogen bonds, hydrogen exodiffusion and Fourier transform infrared spectrometry (FTIR) were performed.

**Figure 9:**
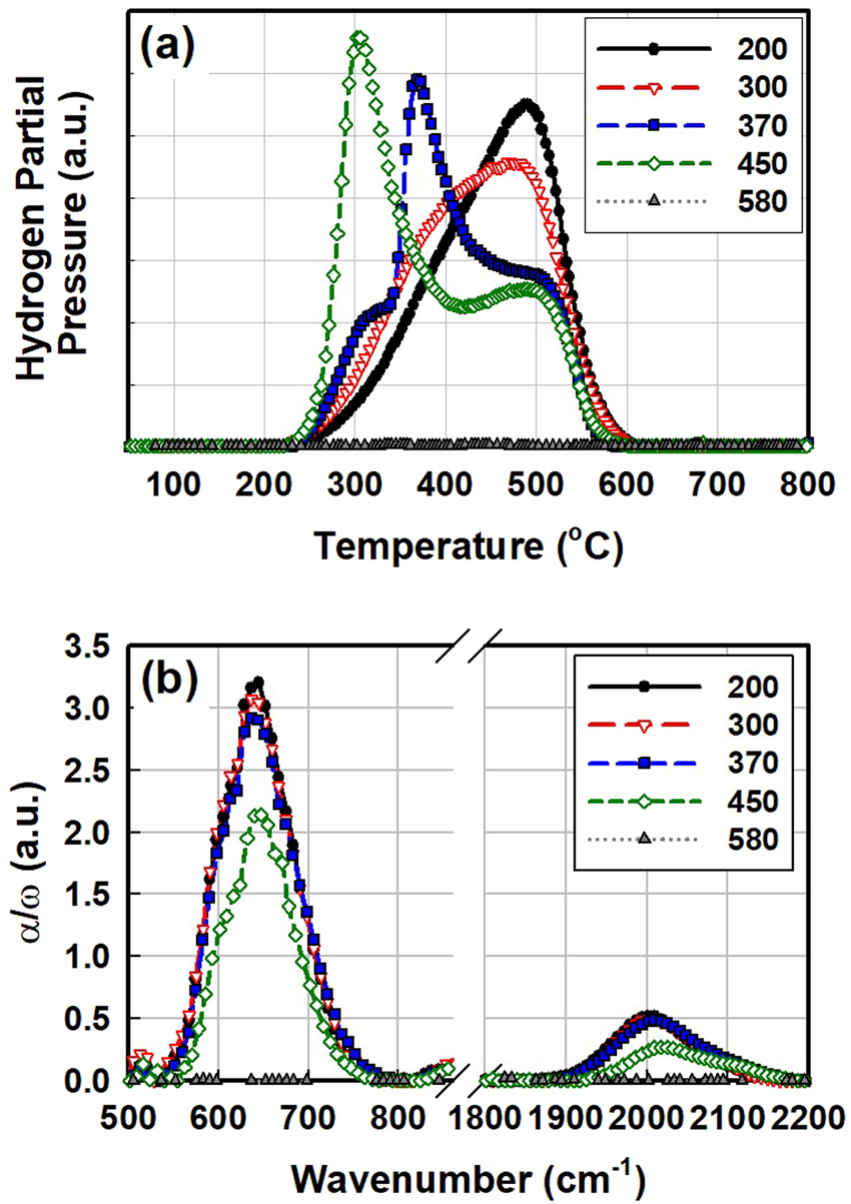
(**a**) Hydrogen exodiffusion result and (**b**) FTIR absorption spectra of a-Si:H fabricated via PECVD at 200, 300, 370, and 450 °C and poly-Si fabricated via LPCVD at 580 °C.

Fig. 9 shows a hydrogen exodiffusion result and FTIR absorption spectra of the substrate-temperature series. Corning glass for hydrogen exodiffusion and <111> FZ c-Si wafer for FTIR were used as the substrates. When a-Si:H is heated at a constant rate, silicon–hydrogen bonds are broken, and the mobile hydrogen effuses. In the hydrogen exodiffusion experiment, a few different hydrogen effusion processes are involved depending on the hydrogen content, sample configuration, and microstructure of the material^27^. At approximately 350 °C, which is relatively low, hydrogen starts to effuse. The low-temperature (LT) peak in Fig. 9 is associated with the rupture of silicon–hydrogen bonds at the internal surfaces of interconnected voids. At the low temperature around 350 °C, the released hydrogen atoms immediately form molecular hydrogen^28^. Furthermore, molecular hydrogen trapped in the nanovoids can be released at a low temperature. Fedders *et al*. reported through nuclear magnetic resonance experiments that up to 40% of the total hydrogen content of a-Si:H is molecular hydrogen^29^. At a higher temperature of approximately 500 °C, silicon–hydrogen bonds in isolated voids start to rupture. In this case, the released hydrogen diffuses in the material in the form of atomic hydrogen; thus, it is a diffusion-rate-limited process. Therefore, the hydrogen effusion process at a high temperature depends on material properties such as thickness and microstructure^30^. Between two hydrogen effusion peaks, a continuous hydrogen effusion signal can be also observed. This signal is attributed to material reconstruction by hydrogen motions^31^ such as hydrogen diffusion and amorphous network relaxation^31,32^.

As shown in Fig. 9(a), the LPCVD sample initially shows no hydrogen evolution except for a background signal due to the substrate. This result is not surprising considering the high substrate temperature of 580 °C and the fact that no blister formation was observed in the optical images shown in Fig. 7. Silane pyrolysis at temperatures above 580 °C completely decomposes the silane molecule into a silicon atom and two hydrogen molecules, and no hydrogen remains in the material. For the a-Si:H sample fabricated via PECVD, hydrogen effusion signals show two trends with respect to substrate temperature. LT effusion peaks shift to lower temperatures for samples deposited at higher substrate temperatures. This result should be attributed to an increase in the internal surface area, i.e., the porosity of the material. Bertran *et al*. analysed hydrogen exodiffusion characteristics of silicon nano powders fabricated via PECVD and observed that the LT effusion peak shifts to a lower temperature when larger nanoparticles are incorporated^33^. Moreover, the intensity of the LT effusion peak appears to be larger at a higher substrate temperature. Furthermore, the intensity of the high-temperature (HT) effusion peaks decrease as the substrate temperature increases. Additionally, the signal level of the HT effusion peak appears to decrease for a sample deposited at a higher temperature.

FTIR can be a good complementary measurement technique to hydrogen exodiffusion because FTIR detects the molecular vibration of silicon–hydrogen bonds, while hydrogen exodiffusion detects the total hydrogen in the material. In FTIR spectra, absorption bands related to the silicon–hydrogen bond are significant at 650 cm^−1^ and 2000 cm^−1^, which are attributed to wagging and stretching modes, respectively^34,35^. Another absorption band is found at approximately 850 cm^−1^, but this band is usually considered less important because of its relatively low signal intensity. The hydrogen content (C_H_) of the material can be deduced by integrating the wagging-mode band at 650 cm^−1^ because the wagging mode corresponds to the total hydrogen content and is independent of the bonding configurations^34,35^. The stretching mode at 2000 cm^−1^ is also important because various bonding configurations, such as monohydride, dihydride, and multihydride (Si-H, Si-H_2_), can be analysed via deconvolution of this band^35,36^.

**Figure 10:**
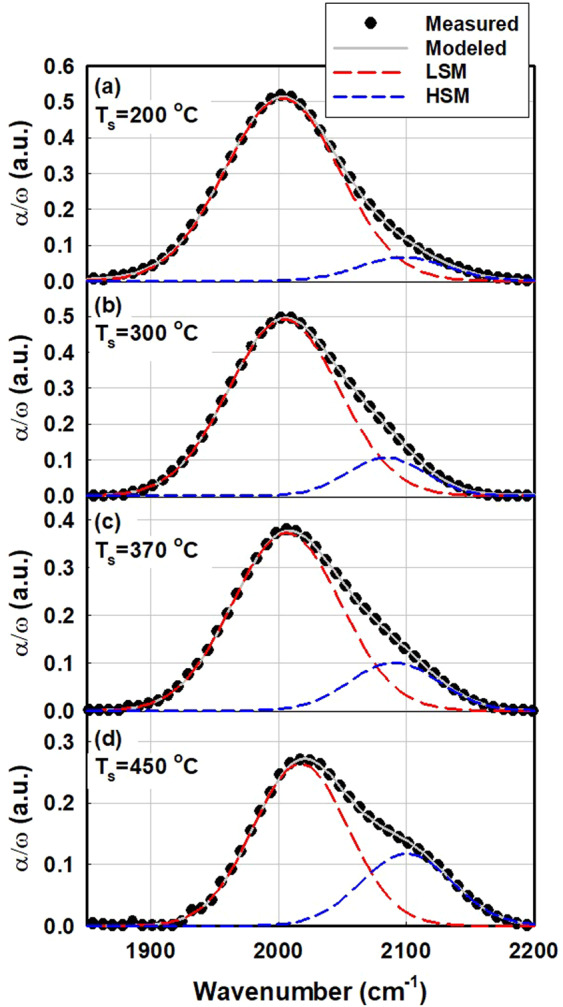
Deconvolution result of stretching mode in FTIR absorption spectra of a-Si:H fabricated via PECVD at 200, 300, 370, and 450 °C in the as-deposited state.

As shown in Fig. 9(b), the poly-Si fabricated via LPCVD shows no absorption bands related to silicon–hydrogen bonds. This result is in good agreement with Figs. 5 and 9(a). The results of this study comprehensively establish that blistering during TOPCon fabrication originates from the rupture of silicon–hydrogen bonds. Therefore, the deposition of a silicon thin film with no hydrogen content should be key to prevent blister formation during TOPCon formation. As mentioned earlier, a low hydrogen content can be achieved by controlling PECVD parameters such as the relative gas flow ratio, resulting in the suppression of blisters^26^. For PECVD samples, the intensities of both the wagging-mode band and stretching-mode band gradually decrease with increasing substrate temperature. Moreover, the dihydride signal of the stretching-mode band at 2100 cm^−1^ is more significant for a sample deposited at a higher substrate temperature, while the integrated area of the entire stretching-mode band decreases as the substrate temperature increases. Stretching-mode absorption peaks of a-Si:H can be analysed using deconvolution to a low stretching mode (LSM) at approximately 2000 cm^−1^ and a high stretching mode (HSM) at approximately 2090–2100 cm^−1^. Figure 10 and Table 1 present the deconvolution result of the stretching-mode band.

**Table 1:** Summary of deconvolution results shown in Fig. 10.

Ts (°C)	I_LSM_ (cm^−1^)	I_HSM_ (cm^−1^)	Microstructure R
200	57.26	5.65	0.089
300	53.21	8.13	0.132
370	39.45	8.8	0.182
450	24.5	10.5	0.299

The ratio of the integrated area of LSM to that of HSM is defined as the microstructure parameter R, which can be used as an indicator of concentration of nanovoids, i.e., the material porosity. The microstructure parameter R is defined as

1 $$R=\frac{{I}_{HSM}}{{I}_{LSM}+{I}_{HSM}},$$

which corresponds to the relative ratio of HSM area to the total stretching-mode area.

**Figure 11:**
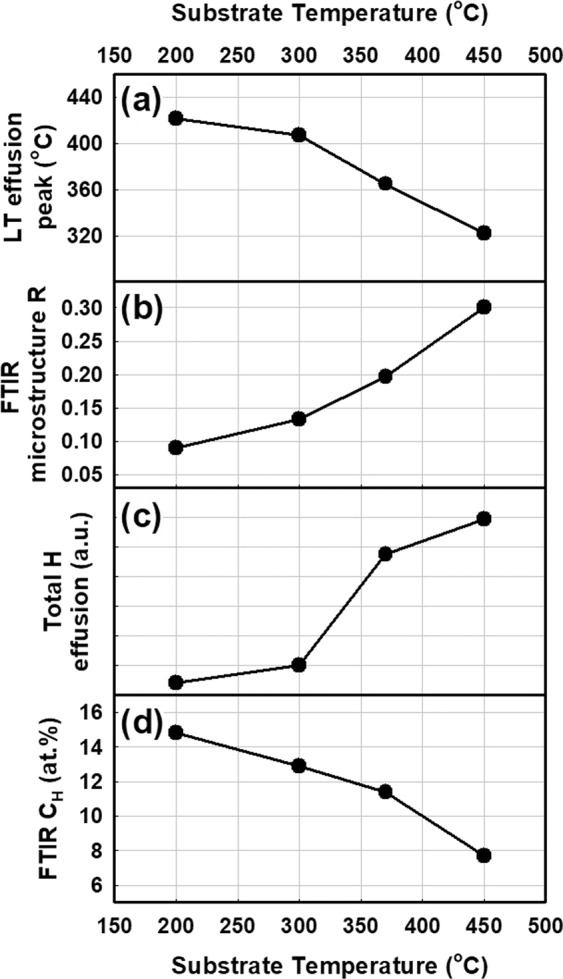
(**a**) Low-temperature hydrogen effusion peak position, (**b**) the microstructure parameter R, (**c**) total hydrogen effusion, and (**d**) the hydrogen content of a-Si:H fabricated via PECVD as functions of the substrate temperature.

Figure 11 shows a representative analysis result of hydrogen in PECVD a-Si:H. The figure plots the position of the LT effusion peak, microstructure parameter R, total hydrogen effusion, and C_H_ as functions of substrate temperature. As the higher substrate temperature during sample deposition increases, the LT effusion peak shifts to a lower temperature, which is attributed to the increase of porosity. Such an increase of porosity is cross verified by the increase of the microstructure parameter R.

Interestingly, as the substrate temperature increases, the total hydrogen effusion increases while C_H_, which is equivalent to the number of silicon–hydrogen bonds, decreases. While hydrogen exodiffusion detects the *total* hydrogen in the sample, either bonded or trapped as molecular hydrogen, FTIR detects *only bonded* hydrogen. Simply put, the increase of substrate temperature from 200 °C to 450 °C caused not only the decrease of hydrogen content, which is unsurprising, but also the increase of molecular hydrogen trapped in voids, which should be markedly taken care.

As mentioned above, at a deposition temperature below 400 °C, the surface of the growing film is covered by hydrogen^22^. The surface coverage of hydrogen causes the surface diffusion of impinging precursor species, resulting in the deposition of a high-quality, dense, and compact material^21^. At a higher temperature, the growing surface would lose hydrogen coverage^25^. Consequently, the impinging precursor species would insert itself into the matrix immediately upon impact with the surface, causing a high sticking probability due to the low mobility. The resulting films would be generally poor in quality and porous with voids^25^. Therefore, even at a substrate temperature below 400 °C, a temperature above that optimised for growth would induce disordered films at a low hydrogen concentration. In other words, in PECVD deposition, although higher temperatures are expected to suppress blistering through the desorption of hydrogen from the surface of the growing film, volume deficiencies of the film, e.g., nanovoids, may ultimately cause the opposite effect at a temperature above the optimum, leading to more severe blistering.

## Conclusion

In this work, we studied the formation mechanism and suppression of blister formation during TOPCon fabrication. PECVD a-Si:H deposited without a tunnelling-oxide layer showed no blister formation, suggesting that the primary reason for blistering is the tunnelling-oxide layer and that blistering can be avoided by removing the tunnelling oxide. However, the tunnelling-oxide layer is necessary to fabricate a passivating contact, and its removal is not a feasible solution to blistering. The investigation of the annealing conditions revealed that the blistered area was always approximately 20% of the optical image surface, irrespective of the annealing temperature and duration. The result led us to conclude that blistering depends on hydrogen effusion during thermal annealing, and blistering should be suppressed by controlling either the interface adhesion or hydrogen content of the thin film.

A TOPCon fabricated on a rough surface showed reduced blister formation after thermal annealing because surface roughness enhanced adhesion. We tested three types of wafer substrates having different surface structures. The fabrication of a TOPCon structure on a KOH-etched solar wafer, showing surface patterns 1–2 μm high and ~50 μm large, resulted in significantly reduced blister formation. Furthermore, the fabrication of a TOPCon structure on a pyramid-textured wafer with a pyramid size of 5 μm resulted in no visible blistering, while that on a CMP-treated semiconductor wafer showed the most significant blistering after thermal annealing.

The other solution is the reduction of hydrogen content in the deposition of the silicon thin film. LPCVD poly-Si at 580 °C was free from blistering after TOPCon formation. Therefore, blistering can be avoided if the silicon thin film is deposited at a high temperature such as 580 °C. A TOPCon deposited at 580 °C indeed showed a passivation quality superior to that of a TOPCon deposited at 200 °C.

Remarkably, the PECVD samples fabricated at 300, 370, and 450 °C showed lower hydrogen contents, but they were already blistered in the as-deposited state. Analysis results indicate that in PECVD deposition, although the increase of substrate temperature reduces the hydrogen content, it risks the increase of porosity and molecular-hydrogen trapping, resulting in even more severe blistering.

In this work, we investigated the passivation property of TOPCon layer stacks through non-contact measurement. However, our work has some limitations, the most significant of which is that a complete solar cell has not been realised using the proposed stacks. Our samples are indeed double-side passivated structures, but several important process steps, such as the boron doping of the front emitter, front surface passivation, and the metallisation of the rear side, should be further developed to realise a complete solar cell. Therefore, further work should be conducted to achieve a high-efficiency crystalline silicon solar cell using a blister-free TOPCon passivating contact.

## Methods

TOPCon layer stacks were fabricated on n-type, 650-μm-thick, 3-ohm∙cm, <100> chemical–mechanical polishing (CMP)-treated semiconductor-grade wafers. n-type, 200-μm-thick, 0.5 ohm∙cm, <100> solar-grade wafers were also used. Samples deposited on the semiconductor-grade wafers were used for optical microscopy, spectroscopic ellipsometry (SE), and measurements of the implied open circuit voltage (implied V_oc_). Samples deposited on the same <100> solar-grade wafers were also used for measurements of implied V_oc_ after thermal annealing. For FTIR measurement, n-type, 650-μm-thick, 5000-ohm∙cm, <111> float-zone wafers were used as the substrate. For hydrogen exodiffusion, Corning Eagle glass was used as the substrate.

The solar-grade wafers were chemically polished using KOH solution and deionised water at 80 °C. Etching was performed to a depth of approximately 10 μm from the wafer surface on both sides. All substrates were cleaned using RCA cleaning in the following sequence: 10% HF dip and deionised water + H_2_O_2_ + NH_4_OH (RCA1) at 80 °C for 10 min, followed by deionised water + H_2_O_2_ + HCl (RCA2) at 80 °C for 10 min and 10% HF dip.

Texturing was done in a mixed solution of 85% KOH: additive: DI water = 0.536 L: 0.281 L: 35 L at 83 °C for 570 s. The height of the resulting pyramids was found to be about 3 μm.

**Figure 12:**
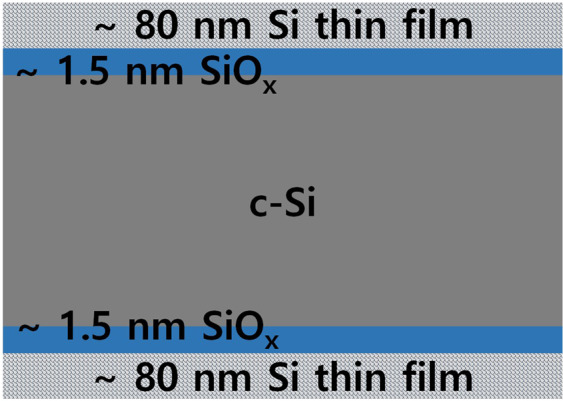
Sketch of sample structure used in this work.

Tunnelling oxide was grown chemically using a nitric acid (HNO_3_) solution. The thickness of the tunnelling-oxide layer was found to be 1.5 nm through SE measurement and modelling. It is known that oxide layers grown using HNO_3_ are usually non-stoichiometric. Details on the HNO_3_ solution can be found elsewhere^37^. On the thin oxide layer, hydrogenated amorphous silicon (a-Si:H) thin films were deposited. In the case of PECVD, a custom-built capacitively coupled plasma (CCP) radio-frequency (RF, 13.56 MHz) glow discharge reactor was used with hydrogen-diluted silane gas mixtures. In this work, our standard a-Si:H layers were deposited at pressures ranging from 0.5 to 1.5 Torr, an RF power density of 30 mW/cm^2^, and a silane–hydrogen dilution ratio of 30:400 at a substrate temperature of 200 °C. The deposition rate of the standard a-Si:H layer was found to be 1.5 Å/s through SE measurement and modelling. In the case of LPCVD, a custom-built single-wafer, cold-wall reactor was used. The LPCVD film was partially crystallised in the polycrystalline form in the as-deposited state. The poly-Si layers were deposited at 100 mTorr with a substrate temperature of 580 °C. The deposition rate of LPCVD was 0.2 Å/s. After thin-film deposition, the wafers were thermally annealed at temperatures ranging from 600 to 1000 °C for 1 or 5 min in a quartz tube furnace under a nitrogen atmosphere. The annealing conditions were identical for PECVD and LPCVD. Figure 12 shows a simple sketch of the sample structure used in this work. Further details of the sample structure can be found in a previous report by our group^15^.

The implied V_oc_ was determined using the quasi-steady-state photoconductance (QSSPC) method using WCT-120 manufactured by Sinton instruments. FTIR measurement was performed using a Shimadzu IRAffinity-1S spectrometer in the transmission mode. Optical images of the TOPCon structure were taken using a Nikon Eclipse LV150N optical microscope. Blisters in the optical images were statistically analysed using ImageJ software (64-bit version, Java 1.8.0_112) to identify blisters and calculate their areas^38^. Hydrogen exodiffusion measurement was performed using a custom-built tool. Samples deposited on Corning glass were loaded in a quartz tube under vacuum (10^−8^ Torr), and the films were heated at a constant ramp rate of 5 °C/min. Effused gas was detected using a Pfeiffer PrismaPro quadrupole mass spectrometer. The sample temperature was measured using a thermocouple, the wires of which were in contact with the sample surface.

## Data Availability

The data that support the findings of this study are available from the corresponding author, [H.S and K.H.K.], upon reasonable request.
